# Whole-Genome Sequencing of Endangered Dengchuan Cattle Reveals Its Genomic Diversity and Selection Signatures

**DOI:** 10.3389/fgene.2022.833475

**Published:** 2022-03-29

**Authors:** Liangliang Jin, Kaixing Qu, Quratulain Hanif, Jicai Zhang, Jianyong Liu, Ningbo Chen, Quji Suolang, Chuzhao Lei, Bizhi Huang

**Affiliations:** ^1^ Yunnan Academy of Grassland and Animal Science, Kunming, China; ^2^ Key Laboratory of Animal Genetics, Breeding and Reproduction of Shaanxi Province, College of Animal Science and Technology, Northwest A&F University, Yangling, China; ^3^ Academy of Science and Technology, Chuxiong Normal University, Chuxiong, China; ^4^ National Institute for Biotechnology and Genetic Engineering, Faisalabad, Pakistan; ^5^ Institute of Animal Science, Tibet Academy of Agricultural and Animal Husbandry Science, Lhasa, China

**Keywords:** whole-genome resequencing, Chinese cattle, genetic diversity, population structure, selection signatures, lactation function

## Abstract

Dengchuan cattle are the only dairy yellow cattle and endangered cattle among Yunnan native cattle breeds. However, its genetic background remains unclear. Here, we performed whole-genome sequencing of ten Dengchuan cattle. Integrating our data with the publicly available data, Dengchuan cattle were observed to be highly interbred than other cattle in the dataset. Furthermore, the positive selective signals were mainly manifested in candidate genes and pathways related to milk production, disease resistance, growth and development, and heat tolerance. Notably, five genes (*KRT39*, *PGR*, *KRT40*, *ESR2,* and *PRKACB*) were significantly enriched in the estrogen signaling pathway. Moreover, the missense mutation in the *PGR* gene (c.190T > C, p.Ser64Pro) showed a homozygous mutation pattern with higher frequency (83.3%) in Dengchuan cattle. In addition, a large number of strong candidate regions matched genes and QTLs related to milk yield and composition. Our research provides a theoretical basis for analyzing the genetic mechanism underlying Dengchuan cattle with excellent lactation and adaptability, crude feed tolerance, good immune performance, and small body size and also laid a foundation for genetic breeding research of Dengchuan cattle in the future.

## Introduction

Chinese domestic cattle breeds have a broad genetic base and abundant genetic variation, generally consisting of *Bos taurus* and *Bos indicus* lineages. *Bos taurus* mainly originated from cattle in Europe and is distributed in northern China, whereas *Bos indicus* originated from cattle in South Asia and is mainly distributed in southern China (Zhang, 2011). Because of the intensive selection, *Bos taurus* have advantages on beef and milk production; however, it is not adapted to tropical environments and thus cannot make use of its full potential for production in hot and humid areas in southern China (Crouse et al., 1989; Buchanan, 2002; Pegorer et al., 2007; Satrapa et al., 2011). Compared to *Bos taurus*, *Bos indicus* is able to tolerate heat and crude feed. One of the typical and distinctive physical features includes a hump on its back (Utsunomiya et al., 2019; Zhang et al., 2021). Scientific research indicates hybridization of these sub-species can combine the strengths of *Bos taurus* and *Bos indicus*, being one of the oldest and truest ways to balance productivity with the environmental adaptability (Negussie et al., 1999; Schatz et al., 2020).

Yunnan has been one of the core regions for the migration of Indian indicine into the Chinese territory. Amongst various hybrid cattle, Dengchuan cattle are the only local dairy yellow cattle breed in China. Dengchuan shares a long history of selective breeding, dating back to the Han Dynasty (206 BC-220). Local people paid attention to selecting cattle with longer lactation periods with high milk yield, to breed their offspring, and using them to make milk fats, which is the local specialty dairy product (Zhang, 2011). With the improvement and promotion of artificial insemination and frozen semen technology of fresh semen at room temperature, native people massively introduced Holstein cattle for hybrid improvement. According to statistics data, from 1981 to 1989, the 305-day milk yield of Dengchuan cattle increased from 838.3 kg to 1,066.6 kg, with a milk fat rate of 6.89% and a dry milk matter of 13.52%, whereas the 305-day milk yield of hybrid F3 (Dengchuan × Holstein) increased from 2,111.1 kg to 3,094.4 kg, with a milk fat percentage of 4.09% and a dry milk matter of 12.0% (Zhang and Ma, 1987; Ma, 1988). However, blind hybridization and the lack of breed conservation planning caused the threat of breed degradation in Dengchuan cattle. According to a recent survey, only 212 Dengchuan cattle (206 cows and six bulls) remained, among which the original breed of Dengchuan cattle is extremely rare and endangered (Yang et al., 2021).

With the development of the next-generation sequencing technology and the enrichment of re-sequencing databases, genome-wide genetic analysis plays an increasingly significant role in the investigation and selection of germplasm resources of landraces (Shen et al., 2020; Jiang et al., 2021; Xia et al., 2021; Zhang et al., 2021). A recent study on Dengchuan cattle showed that Dengchuan cattle are a taurine–indicine mixed breed (Foissac et al., 2019). However, there are no previous studies using whole-genome sequencing data to identify genes related to milk production and disease resistance in Dengchuan cattle.

In this study, we performed whole-genome sequencing of ten individuals of Dengchuan cattle to explore the genetic diversity and population genetic structure of the autosomal genome. In order to further explore the genetic potential of Dengchuan cattle, single nucleotide polymorphisms (SNPs) of Dengchuan cattle were compared with those of commercial and native breeds previously collected from around the world.

## Materials and Methods

### Sample Collection and Sequencing

Ten samples of Dengchuan cattle were collected from the ear tissue samples in the Dengchuan area of Yunnan province, China. To explore the ancestry proportions of Dengchuan cattle and compare the genetic diversity with worldwide cattle breeds, additional 68 samples were collected from the Sequence Read Archive (SRA, https://www.ncbi.nlm.nih.gov/sra/) (Leinonen et al., 2011), including European cattle breeds [Angus (n = 9), Simmental (n = 8), and Holstein (n = 8)]; northeast Asia breed (Hanwoo, n = 10); southwest Chinese breeds [Dengchuan (n = 2), Dianzhong (n = 6), and Wenshan (n = 6)]; southeast Chinese breeds [Guangfeng (n = 4) and Wannan (n = 5)]; and India–Pakistan zebu cattle (*Bos indicus*) breeds (n = 10) (Supplementary Table S1). A total of 78 samples were used in this study.

### Sequencing, Alignment, and Variant Identification

Genomic DNA was extracted using the standard phenol–chloroform method (Sterky et al., 2017). Paired-end libraries with the average insert size of 500 bp were constructed for each individual, with an average read length of 150 bp and an average sequence coverage of ∼10.7×. Sequencing was performed using Illumina NovaSeq instruments at Novogene Bioinformatics Institute, Beijing, China. Raw reads data of fastq format were quality trimmed using trimmomatic (SLIDINGWINDOW:3:15 MINLEN:35 TRAILING:20 LEADING:20 AVGQUAL:20 TOPHRED33) (Bolger et al., 2014) to remove adapters and low-quality bases. The Burrows–Wheeler Aligner BWA-MEM (v0.7.15-r1140) with default parameters (Li and Durbin, 2009) was used to align the clean reads to the *Bos taurus* reference assembly ARS-UCD1.2. The Picard tools (http://broadinstitute.github.io/picard) were used to filter potential duplicate reads. We used “Haplotype Caller,” “Genotype GVCFs,” and “Select Variants” modules of the Genome Analysis Toolkit (GATK, version 3.8-1-0-gf15c1c3ef) (McKenna et al., 2010) to call the SNP. The filtration of raw SNPs was conducted by using “variant Filtration” modules with the parameters “QD < 2.0, FS > 60.0, MQ < 40.0, MQRankSum < −12.5, ReadPosRankSum < −8.0, and SOR > 3.0” and the mean sequencing depth of variants (all individuals) “<1/3× and >3×”. Based on the *Bos taurus* reference assembly ARS-UCD1.2, SNPs were functionally annotated by ANNOVAR (Wang et al., 2010).

### Population Genomic Analysis

SNPs of 78 samples were pruned in high levels of pairwise LD by PLINK v1.90b3.40 software (Purcell et al., 2007), excluding SNPs in strong LD (r2 > 0.2) within a sliding window of 50 SNPs advanced by five SNPs at the time. Principal component analysis (PCA) was carried out using the smartpca program of the EIGENSOFT v5.0 package (Patterson et al., 2006). Population structure analysis was carried out by ADMIXTURE v1.3.0 (Alexander and Lange, 2011). Based on the pairwise distance matrix, the NJ tree was constructed by MEGA v10.2.6 (Saitou and Nei, 1987; Kumar et al., 2018).

Runs of homozygosity (ROHs) were calculated by PLINK software (Purcell et al., 2007). SNPs with minor allele frequencies (MAF) < 0.05 were excluded due to instability. PLINK uses a sliding window of a minimum of 50 SNPs across the genome to identify ROHs, allowing for two missing SNPs and one heterozygous site per window. The minimum number of continuous homozygous SNPs constituting an ROH was set to 100. The minimum SNP density coverage was set to at least 50 SNPs per Kb, allowing for centromeric and SNP-poor regions to be algorithmically excluded from the analysis. The maximum gap between two consecutive homozygous SNPs was set at 100 Kb. The number and length of ROHs for each breed were estimated, and the length of ROH was divided into three categories: 0.5–1 Mb, 1–2 Mb, and 2–4 Mb, reflecting ancient, historical, and recent inbreeding, respectively (Kirin et al., 2010; Bhati et al., 2020).

Nucleotide diversity of each breed was investigated by VCFtools (Danecek et al., 2011) with the size of 50-kb non-overlapping window. The output of the --het function by VCFtools is a summary for each individual of the observed number of homozygous sites (O(hom)) and the expected number of homozygous sites (E(hom)). It also includes the total number of sites that the individual has data for and the inbreeding coefficient F, which is the canonical estimate of genomic F based on excess SNP homozygosity (Keller et al., 2011).

Linkage disequilibrium (LD) decay with the physical distance between SNPs was calculated and visualized by PopLDdecay software (Zhang et al., 2019) with default parameters.

### Selective Sweep Identification

Only SNPs with less than 10% missing were used for selective sweep scanning. The nucleotide diversity (θπ) and the composite likelihood ratio (CLR) test (Nielsen et al., 2005) were used to detect the selection signatures in Dengchuan cattle and Holstein cattle. The θπ was estimated based on a sliding window of size 50 kb and a step of size 20 kb by VCFtools (Danecek et al., 2011). The CLR was calculated for sites in non-overlapping 50-kb windows by SweepFinder2 (DeGiorgio et al., 2016), reflecting the likelihood of observing SNP data under the assumption of a sweep.

We also performed the genetic differentiation (*F*_ST_) and cross-population composite likelihood ratio test (XP-CLR) (Chen et al., 2010) to identify the difference in potential areas between different cattle breeds. *F*_ST_ analysis was estimated based on a sliding window of 50 kb and a step of size 20 kb by VCFtools (Danecek et al., 2011). XP-CLR is a likelihood method for detecting selective sweeps by jointly modeling the multilocus allele frequency differentiation between the two groups (Chen et al., 2010). The overlap of the top 1% window in each method was considered as candidate signatures of selection, and genes in those window regions were defined as potential candidate genes.

### Enrichment Analyses of Candidate Genes Under Selection

Due to the complexity of biological data-mining situations, enrichment analysis was conducted to identify the possibility of biological processes associated with Dengchuan cattle (Huang et al., 2009). Online Kyoto Encyclopedia of Genes and Genomes (KEGG) pathway and Gene Ontology (GO) analyses were conducted by KOBAS 3.0 (Bu et al., 2021). Genes at *p* < 0.05 were considered to be significantly enriched in Kyoto Encyclopedia of Genes and Genomes (KEGG) pathways and GO (Gene Ontology) annotations.

### Aligning Candidate Regions to the Quantitative Trait Loci Database

Biological processes of Dengchuan cattle could be analyzed through genes annotated from candidate regions; however, there were various candidate regions in the non-annotated genic regions, although they showed strong selective signals. We used QTLs to identify possible traits in these candidate regions. The cattle QTL database (http://www.animalgenome.org/cgi-bin/QTLdb/BT/index) contains 163,725 QTLs. The chromosome information is annotated to the cattle QTLdb to identify the regions of interest detected by selective sweep methods contained or overlapped across the QTLs. The function and information of candidate regions were determined after annotation.

## Results

### Analysis of the Population Structure and Genetic Diversity

**FIGURE 1:**
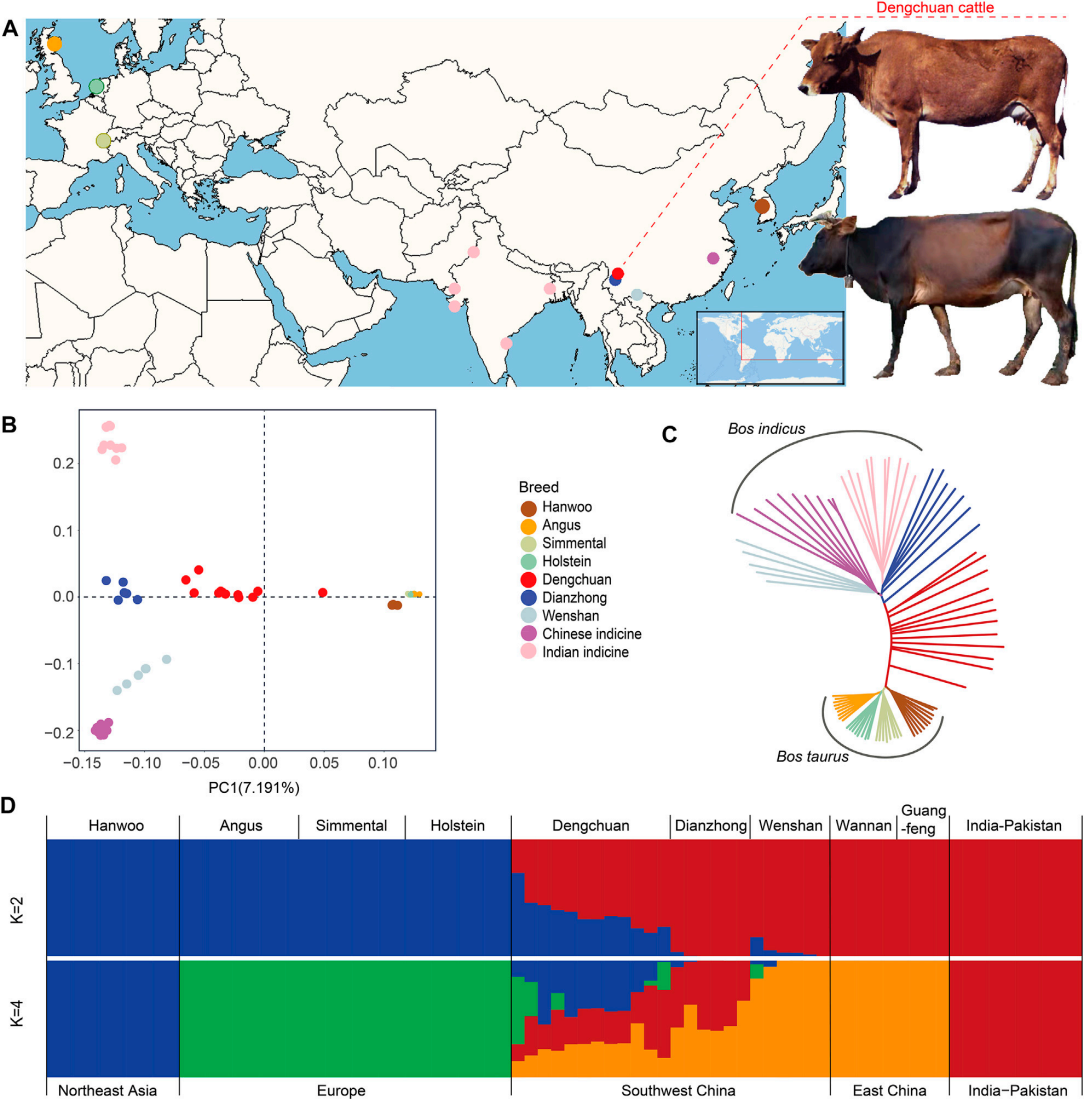
Population genetic analysis of Dengchuan cattle. **(A)** Geographic map indicating the origins of Dengchuan cattle and other cattle analyzed in this study. **(B)** Principal component analysis of cattle with PC1 against PC2. **(C)** Neighbor-joining tree of the 78 domesticated cattle. **(D)** Admixture plot (K = 2, 4) for the 78 cattle individuals. Each individual is shown as a vertical bar divided into K colors.

Seventy-eight cattle, representing five geographically diverse cattle populations, namely, East Asian taurine, European taurine, Chinese indicine, Indian indicine (Xu et al., 2018), and local hybrid populations in Yunnan were selected for genome re-sequencing analysis (Figure 1A, Supplementary Table S1). After quality control, 147,397,064 bi-allelic autosomal SNPs (Supplementary Table S2) were used to construct genetic relationships using a neighbor-joining maximum likelihood method and PCA. Both methods revealed that these populations of regions, except Yunnan, clustered into three major genetic groups: *Bos taurus*, Indian indicine, and China indicine (Figure 1B, C). It was clear that Dengchuan cattle and Dianzhong cattle showed a certain degree of hybridization. Admixture analysis showed that the cattle breeds separate into *Bos taurus* and *Bos indicus* ancestries (Figure 1D, K = 2). When the number of clusters (K) was set to 4, East Asian taurine and European taurine were clearly separated. Dengchuan cattle depicted clear evidence of genetic heterogeneity with its shared genome ancestry with East Asian taurine (Hanwoo), European taurine (Angus, Simmental, and Holstein), Chinese indicine (Wannan and Guangfeng), and Indian indicine. It is rather remarkable that only half the Dengchuan cattle had a European taurine ancestry (Figure 1D, K = 4).

### Patterns of Genomic Variation

**FIGURE 2:**
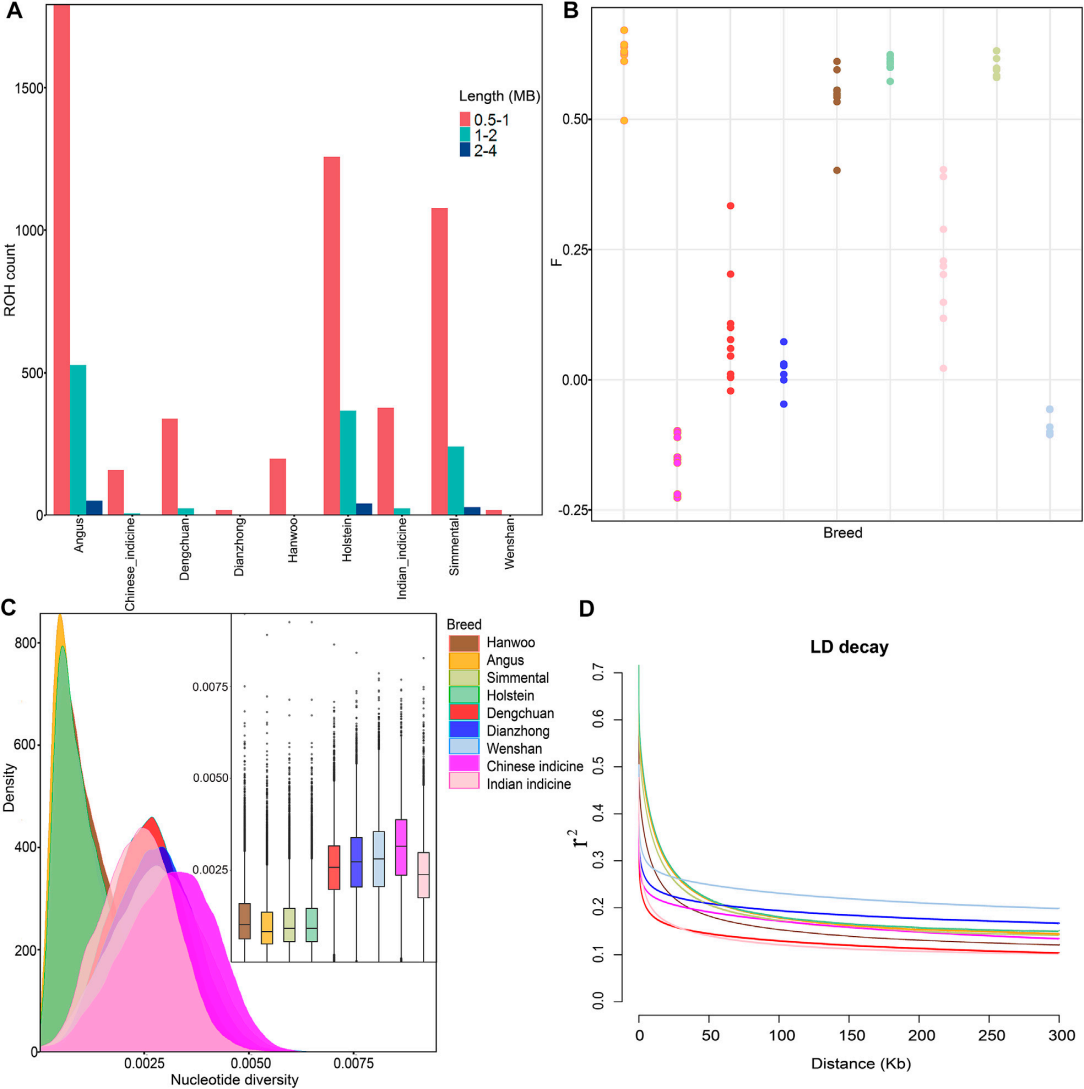
Genetic diversity among 78 samples from nine populations. **(A)** Estimation of the total number of ROH for each group. The three categories of ROH length: 0.5–1 Mb, 1–2 Mb, and 2–4 Mb, reflecting ancient, historical, and recent inbreeding, respectively. **(B)** Inbreeding coefficient for each individual. **(C)** Density plots and Box plots of the nucleotide diversity for each group. **(D)** Genome-wide average LD decay estimated from each group.

ROH analysis revealed that the vast majority of ROHs identified in all breeds were between 0.5–1 Mb in length, but European commercial breeds (Angus, Holstein, and Simmental) had medium (1–2 Mb) and long ROHs (2–4 Mb). Besides, the total lengths of ROHs in Dengchuan cattle were much longer than those of the other two cattle in Yunnan (Figure 2A). This could indicate that European commercial breeds and Dengchuan cattle had undergone artificial selection for a long time. Similarly, the inbreeding coefficient based on genome heterozygosity was the highest in Angus (0.67) and lowest in Chinese indicine (−0.22) (Figure 2B). The average nucleotide diversity among Dengchuan cattle and other cattle groups revealed that Chinese indicine was the highest (3.10e−3), followed by Wenshan cattle (2.81e−3), Dianzhong cattle (2.72e−3), and Dengchuan cattle (2.58e−3). In comparison to *Bos indicus*, it was concluded that *Bos taurus* possessed a low level and high density of nucleotide diversity (Figure 2C). In contrast, the lowest average genome-wide LD was observed in Dengchuan cattle and Indian indicine. Besides, the LD decay in *Bos indicus* was faster than *Bos taurus* when the physical distance of SNP was less than 10 KB (Figure 2D).

### Positive Selective Signature

**FIGURE 3:**
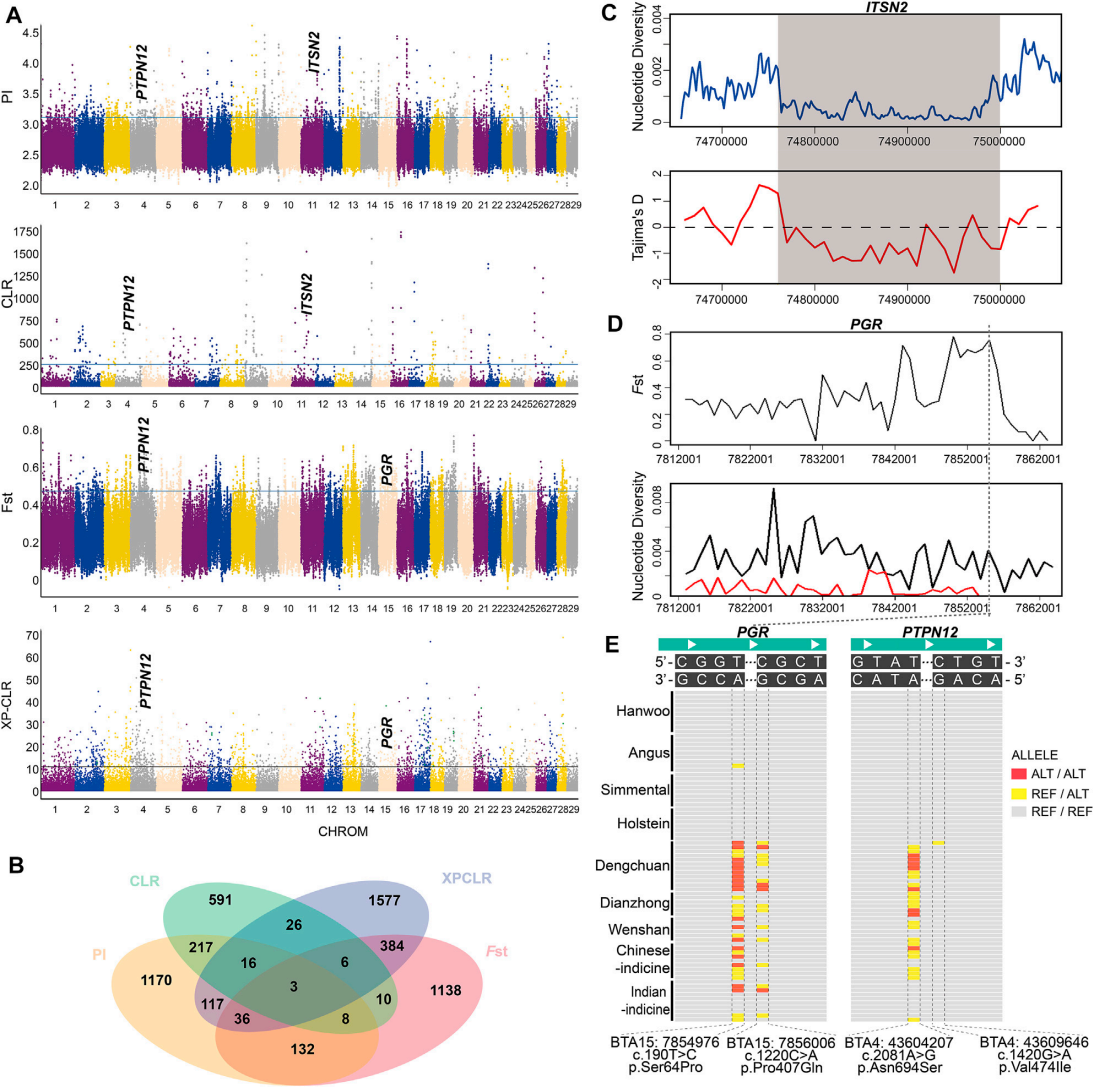
Analysis of the signatures of positive selection in the genome of Dengchuan cattle. **(A)** Manhattan plot of selective sweeps in Dengchuan cattle. **(B)** Venn diagram showing the gene overlaps among θπ, CLR, *F*st, and XP-CLR. **(C)** Nucleotide diversity and Tajima’s D plots at the *ITSN2* gene region. **(D)** *F*st and Tajima’s D plots at the *PGR* gene region. **(E)** Regional highlight of the missense mutation of genes enriched in the estrogen signaling pathway. *PGR* gene showed a high-frequency homozygous mutation site (c.190T > C) in Dengchuan cattle.

A total of 217 candidate genes were detected by both θπ and the CLR test in Dengchuan cattle (Figure 3A, Supplementary Tables S3, S4). Some positively selected genes were reported to be associated with lactation function and disease resistance, such as the butterfat rate [*PPARGC1A* (Weikard et al., 2005; Schennink et al., 2009)], milk production [*B4GALT1* (Asadollahpour Nanaei et al., 2020; Valsalan et al., 2021)], immunity [*IL2* (McCoard et al., 2019) and *NFATC3* (Hu et al., 2018)], and mastitis resistance [*ITSN2* (Miles and Huson, 2020)]. In particular, *ITSN2* was located at the strongest selection signal on BTA11 (11:74700001-74900000). The result of strong positive selection was further verified by Tajima’s D and nucleotide diversity analysis (Figure 3C). Moreover, 217 candidate genes were compared with 224 candidate genes detected by both θπ and the CLR test in Holstein cattle (Supplementary Tables S5, S6), whereas seven genes (*RERE*, *SLC45A1*, *RAB11FIP2*, *PTDSS1*, *MTERF3*, *KDM4C*, and *COL27A1*) were shared in both Dengchuan cattle and Holstein cattle.

### Biological Process and Pathway Between Dengchuan Cattle and Angus Cattle

*F*_ST_ and XP-CLR tests were performed to detect the positive selection signatures between Dengchuan and Angus cattle (Figure 3A, Supplementary Tables S7, S8). A total of 384 genes were overlapped by both methods, which were enriched using GO annotation and KEGG pathway terms to further analyze their biological functions. The results represented significant enrichment of 220 GO terms and 29 KEGG pathways (*p* < 0.05; Supplementary Tables S9, S10). Gene list analysis revealed the involvement of various genes in protein synthesis (GO:0042802), endoplasmic reticulum (GO:0005783), temperature homeostasis (GO:0001659), and neutral amino acid transport (GO:0015804). Similarly, the enriched KEGG pathways included the estrogen signaling pathway, protein processing in the endoplasmic reticulum, biosynthesis of amino acids, glycan biosynthesis, and metabolic pathways. Moreover, *PGR*, a gene enriched in the estrogen signaling pathway, showed strong positive selection in Dengchuan cattle (Figure 3D).

It is worth noting that three overlapped genes (*PTPN12*, *KIAA1109*, and *ADAD1*) were detected among the four mentioned selection methods (Figure 3B), indicating that these genes were strongly selected in Dengchuan cattle. We checked mutations of eight genes (five estrogen signaling pathway enrichment genes (*KRT39*, *PGR*, *KRT40*, *ESR2,* and *PRKACB*) and three overlapped genes (*PTPN12*, *KIAA1109*, and *ADAD1*)) in Dengchuan cattle, two missense mutations in *PGR* (c.190T > C, p.Ser64Pro; c.1220C > A, p.Pro407Gln), and two missense mutations in *PTPN12* (c.2081A > G, p.Asn694Ser; c.1420G > A, p.Val474Ile), which showed distinct allelic patterns in Dengchuan cattle (Figure 3E).

### QTLs Based on Identified Regions

QTLs and selection signatures at the same location indicated that phenotypes and traits were influenced by the joint action of large numbers of polygenes and environmental effects (Georges et al., 1995). Thus, the top 50 candidate regions were studied for each method of scanning in Dengchuan cattle. These candidate regions also included regions that annotated gene failures and were used to extract relevant QTLs from the cattle QTLdb. Since the candidate region might overlap with several QTLs associated with different traits, one result with the most consistent chromosome fragment for each candidate region was picked. As shown in the Supplementary Table S11, 94 genomic regions for 100 candidate regions (θπ and CLR) overlapped with QTLs: 49 candidate regions overlapped milk, 21 candidate regions overlapped reproduction and production, 18 candidate regions overlapped health, and six candidate regions overlapped meat and carcass. Simultaneously, 99 genomic regions for 100 candidate regions (*F*_ST_ and XP-CLR) overlapped with QTLs: 50 candidate regions overlapped milk, 38 candidate regions overlapped reproduction and production, six candidate regions overlapped meat and carcass, four candidate regions overlapped health, and one candidate region overlapped exterior conformation (Supplementary Table S12).

## Discussion

Genomic information is the instruction of life construction. Here, we have conducted the whole-genome sequence-based study for the genomic diversity and selective signatures in Dengchuan cattle. The ancestral contributions of Dengchuan cattle came from East Asian taurine (∼34%), Chinese indicine (∼22%), European taurine (10%), and Indian indicine (∼34%). It is worth noting that Dengchuan cattle have not been able to cluster completely, showing some differences amongst its individuals (Figure 1B). Similar outliers can be seen for the inbreeding coefficient (F) based on ROH, which may be from hybrid lineages or the introduction of crossbreeding. In addition, the ROH distribution and nucleotide diversity of Dengchuan cattle were basically consistent with other native Yunnan breeds (Foissac et al., 2019; Zhang et al., 2021). The LD decay pattern of Dengchuan cattle was similar to that of Indian indicine, confirming the high genetic diversity of Dengchuan cattle.

Dairy cows in hot and humid areas are naturally more prone to environmental mastitis due to bacterial growth (Alain et al., 2009). *ITSN2* is a member of a family of proteins involved in clathrin-mediated endocytosis that encodes a cytoplasmic protein which contains SH3 domains. *ITSN2* is thought to regulate the formation of clathrin-coated vesicles and may also function in the induction of T-cell antigen receptor (TCR) endocytosis (National Center for Biotechnology Information, 2017). Furthermore, *PTPN12* is a protein tyrosine phosphatase that contributes to the stable 3D acinar formation of mammary epithelial cells (Sun et al., 2011). *PGR* promotes alveologenesis in the pregnant mammary gland for milk production (Aikawa et al., 2020). In addition, among the seven genes overlapped between Dengchuan cattle and Holstein cattle, we examined the scanning signal of *PTDSS1* and found that the CLR value in Dengchuan cattle (∼638) was much higher than that in Holstein cattle (∼431). *PTDSS1* is used for the catalytic synthesis of lecithin. The remaining six genes were also reported to be highly associated with milk production: *RERE* and *SLC45A1* are reported to be associated with milk production (Buaban et al., 2022); *MTERF3* is associated with milk fatty acid composition (Palombo et al., 2018); *KDM4C* is associated with breast cancer (Garcia and Lizcano, 2016); *RAB11FIP2* is associated with transcytosis (Ducharme et al., 2007); and *COL27A1* is associated with the sternum (Maddirevula et al., 2019). The strong selection of these genes may be the reason why Dengchuan cattle are a good dairy breed in hot and humid climates.

The comparative analysis of genetic differentiation between Dengchuan cattle and other breeds revealed the estimated value of Dengchuan cattle and Angus cattle to be the highest (∼0.22), which was suitable for subsequent analysis (Supplementary Table S13). Interestingly, the KEGG pathway with significant enrichment of differential signals included the estrogen signaling pathway. Activation of the estrogen signaling pathway results in prolonged lactation and high milk yield (Lawrence, 2022). Additionally, in the current study, the missense mutations in PGR (c.190T > C, p.Ser64Pro) (Figure 3E) likely play an important role in elevated milk production in Dengchuan cattle.

Milk yield and composition are typical polygenic traits (Georges et al., 1995). For a more comprehensive explanation of the function in strong candidate regions, the most promising QTLs were matched for two-hundred candidate regions. Because of the window size of scanning methods and the different fragment sizes of different genes and QTLs, some regions were separated and calculated several times and were matched to the same QTL. This results in a large number of milk-related QTLs matching our candidate regions. Overall, about half of the candidate regions matched QTLs associated with milk yield and its composition. Furthermore, the candidate regions corresponded with the health QTLs, which were mainly related to the somatic cell score and heat tolerance. Most of the QTLs related to reproduction were associated with the body size in our result. For example, some growth-related QTLs have been annotated on chromosome 14 (Supplementary Table S11). Similarly, the *PLAG1* gene on chromosome 14 has been shown to be associated with the body size (Karim et al., 2011). *F*st results showed that there exists significant genetic differentiation of the *PLAG1* gene between Dengchuan cattle and Angus cattle (Supplementary Table S7). It is worth noting that Dengchuan cattle are small in body stature, approximately 105 cm tall and weighing 225 kg in adulthood (Zhang, 2011).

In conclusion, this study provides a theoretical basis for analyzing the genetic mechanism of Dengchuan cattle with excellent lactation and adaptability, crude feed tolerance, good immune performance, and small body size, which also lays a foundation for genetic breeding research of Dengchuan cattle in the future.

## Data Availability

The datasets presented in this study can be found in online repositories. The names of the repository/repositories and accession number(s) can be found in the article/Supplementary Material.
